# Medication Adherence Measurement in Southeast Europe: A Comparative Analysis of Assessment Methods, Digital Health Implementation, and Regulatory Frameworks

**DOI:** 10.3390/healthcare14152419

**Published:** 2026-08-06

**Authors:** Dijana Miceva, Răzvan Nicolae Rusu, Veronica Bild, Marina Odalović, Tanya Kazakova, Anna Todorova

**Affiliations:** 1 Faculty of Medical Sciences, Goce Delcev University, Krste Misirkov Str., No. 10-A, P.O. Box 201, 2000 Stip, North Macedonia; dijana.miceva@ugd.edu.mk; 2 Discipline of Pharmacodynamics and Clinical Pharmacy, Faculty of Pharmacy, Grigore T. Popa University of Medicine and Pharmacy, Universitatii Street No. 16, 700115 Iasi, Romania; razvan-nicolae.rusu@umfiasi.ro (R.N.R.); veronica.bild@umfiasi.ro (V.B.); 3 Faculty of Pharmacy, University of Belgrade, 11000 Belgrade, Serbia; modalovic@pharmacy.bg.ac.rs; 4 Faculty of Pharmacy, Medical University Varna, 9000 Varna, Bulgaria; anna.todorova@mu-varna.bg

**Keywords:** medication adherence, medication adherence tools, assessment methods, regulatory framework, pharmaceutical care, digital health technologies, electronic prescribing, community pharmacy services

## Abstract

Medication adherence is a critical determinant of treatment effectiveness; however, its measurement and integration into routine healthcare practice remain challenging in many healthcare systems. This study aimed to compare medication adherence measurement methods and examine the legal and regulatory frameworks governing adherence-related tools in North Macedonia, Romania, Serbia, and Bulgaria. A structured comparative, two-phase document review was conducted between January and April 2026. The first phase identified and compared medication adherence measurement methods reported in research and recognized within national healthcare systems. The second phase analyzed legislation, regulatory documents, and policy frameworks relevant to adherence-related tools, including digital technologies. Searches were performed in PubMed, Scopus, and Web of Science, together with official governmental, regulatory, and institutional websites. The final evidence base comprised 84 documents, including scientific publications, official legal and policy documents, and regulatory and institutional sources. Across North Macedonia, Romania, and Bulgaria, medication adherence monitoring was not systematically integrated into routine clinical practice, despite differences in digital health infrastructure and regulatory maturity. Traditional indirect methods, particularly prescription refill data and clinician assessment, were more consistently reported, whereas validated direct and digital adherence measurement approaches remained unevenly implemented. Regulatory frameworks differed substantially in scope, specificity, and formal recognition of adherence-related tools, reflecting varying levels of alignment with European digital health policies and contributing to fragmented implementation across healthcare systems. Although existing digital health infrastructure provides a foundation for systematic medication adherence monitoring, implementation remains fragmented because of limited interoperability, inconsistent regulatory support, and the absence of standardized national approaches. Strengthening regulatory guidance, harmonizing adherence measurement standards, integrating adherence indicators into routine healthcare information systems, and formally supporting pharmacist-led adherence services could support more consistent medication adherence monitoring and improve medication management across the healthcare systems of the four analyzed countries.

## 1. Introduction

Medication adherence (MA) is a widespread problem affecting all stakeholders in healthcare; however, responsibility for addressing it remains insufficiently defined [1]. On average, adherence to prescribed therapies is approximately 50%. The World Health Organization (WHO) defines medication adherence as “the degree to which an individual’s behavior, including taking medication, following a diet, and making lifestyle changes, corresponds with the agreed recommendations from a healthcare provider” and recognizes adherence as a multidimensional process influenced by interacting patient-, condition-, treatment-, socioeconomic-, and healthcare system-related factors [2].

One of the main challenges in medication adherence research is the lack of standardized definitions and measurement approaches, which limits the comparability of findings across studies [3].

Medication adherence can be conceptualized at two complementary levels: the individual patient level, where adherence reflects the extent to which a patient’s medication-taking behavior corresponds with the agreed therapeutic recommendations, and the population level, where adherence is aggregated across individual patients to evaluate adherence patterns, disparities, and healthcare system performance.

To provide a standardized conceptual framework, Vrijens et al. proposed the Ascertaining Barriers for Compliance (ABC) taxonomy, which defines medication adherence as a dynamic process comprising three measurable components: initiation, implementation and persistence [4]. The first component, initiation, refers to the point at which a patient takes the first prescribed dose of medication. Implementation describes the extent to which the patient’s actual dosing corresponds to the prescribed regimen from treatment initiation until the last dose taken. Persistence represents the duration of time from treatment initiation until treatment discontinuation, including the occurrence of clinically relevant treatment gaps.

Two additional distinctions are important for adherence measurement. Primary adherence refers to whether a newly prescribed medication is ever dispensed or treatment is initiated, whereas secondary adherence reflects whether patients continue to refill and take their medication as prescribed after initiation. Most quantitative adherence measures evaluate secondary adherence [5].

Several international organizations have incorporated standardized adherence measures into healthcare quality evaluation. For example, in the United States, the National Committee for Quality Assurance (NCQA) and the Pharmacy Quality Alliance (PQA) recommend pharmacy refill-based measures, particularly the Proportion of Days Covered (PDC), as a quality indicator for medication adherence in chronic diseases. These standardized approaches facilitate benchmarking across healthcare systems and support the development of evidence-based adherence improvement strategies [6,7].

MA can be assessed using multiple measurement methods, and a wide range of interventions have been developed and implemented at different levels. Adherence measurement methods are generally categorized into direct and indirect approaches. Direct methods involve objective assessment of medication intake, including directly observed therapy, detection of the drug or its metabolites through biological assays, and ingestible sensor technologies. In contrast, indirect methods assess adherence through patient behavior or medication availability data and include self-reported adherence questionnaires, pill counts, pharmacy refill records, healthcare claims analysis, and electronic medication monitoring systems [8,9,10,11,12].

Emerging digital monitoring technologies show potential to improve adherence and long-term health outcomes [13,14]. Adherence remains a challenge in self-managed treatments, and expanding our understanding of patient barriers is essential to develop more effective interventions and to improve health outcomes. Medication adherence measurement methods and adherence-promoting interventions represent distinct concepts: while measurement methods aim to quantify or characterize medication-taking behavior, including initiation, implementation or persistence, adherence-promoting interventions intend to improve that behavior and can include patient education, medication review, reminders, regimen simplification or pharmacist counseling [9,15]. Guidance exists to support healthcare professionals in delivering adherence-promoting services, and numerous examples of effective practices—such as patient education, medication regimen management, clinical pharmacist consultations, combination pills, and medication reminders—can be replicated and adapted across healthcare settings. However, widespread implementation, funding, and integration of adherence support services remain limited. A large cross-sectional survey of healthcare professionals (HCPs) from 37 European countries showed that digital technological solutions were among the least utilized strategies, while medication review and therapy optimization were more frequently implemented in Western Europe [16]. The choice of intervention should consider feasibility within the health system and the specific challenges faced by patients, highlighting the importance of policy measures for sustainable improvements in adherence [17,18,19].

Smart digital adherence tools can facilitate real-time data collection of medication intake, enabling healthcare providers to identify non-adherence and to make timely data-driven recommendations to their patients (smart bottle sensor data and stakeholder valuation) [20]. A recent systematic review and meta-analysis of 26 randomized controlled trials found that mobile interventions integrated with electronic prescription had significant effects on improving medication adherence [21]. Another systematic review included 12 randomized controlled trials that revealed that most interventions targeted chronic diseases and commonly incorporated features such as medication reminders, educational content, two-way communication, and gamification, which improved adherence [22].

However, the successful implementation and sustainable integration of these digital solutions into clinical practice depend not only on technological innovation but also on the regulatory and policy environment in which they operate. Policies and legislation play a crucial role in ensuring safety, transparency, and public trust in digital health technologies. Regulatory frameworks establish standards for data protection, interoperability, ethical use of health information, and clinical validation.

At the European Union level, Regulation (EU) 2025/327 established the European Health Data Space (EHDS), providing a common legal and technical framework for access to electronic health data. The regulation also establishes interoperability requirements for electronic health record systems and provides for the progressive cross-border exchange of priority health-data categories, including electronic prescriptions and electronic dispensations. Although the EHDS Regulation does not establish a harmonized medication-adherence measure, interoperable prescribing and dispensing data could provide an infrastructural basis for the development and implementation of standardized adherence indicators [23].

The availability of adherence measurement methods in middle-income and upper-income countries, including North Macedonia, Serbia, Bulgaria, and Romania, remains limited, and interventions are often not implemented or supported by legislation.

As a result, assessing adherence accurately is challenging. The limited implementation and integration of validated digital adherence monitoring tools underscores the need for comprehensive evaluation of their feasibility, clinical utility, and potential for broader adoption within healthcare systems. The study’s specific contribution is to synthesize evidence showing that adherence measurement is still fragmented, study-specific, and unevenly implemented, rather than integrated into routine national practice.

Prior work shows isolated tool validation or local use, but not a mature, system-wide implementation model across these countries. This matters because the countries differ not only in regulatory frameworks, but also in the availability of tools, validation of instruments, and real-world uptake. The aim of this study was to conduct a structured comparative review of medication adherence measurement methods and the regulatory frameworks governing their implementation in Bulgaria, Romania, Serbia, and North Macedonia. The research question was as follows: What medication adherence measurement methods are currently available in these countries, and how do the national legal and regulatory frameworks support their implementation in healthcare practice?

## 2. Materials and Methods

### 2.1. Study Design

This study applies a structured comparative documentary review design with thematic content analysis to examine medication adherence measurement methods and the legal and regulatory frameworks that shape their implementation across Bulgaria, Romania, Serbia, and North Macedonia. These countries were purposively selected to provide a shared Southeast European context while varying in regulatory alignment and implementation arrangements, which supports structured cross-country comparison. This comparison also permits analysis of two European Union Member States, Bulgaria and Romania, alongside two EU candidate countries, Serbia and North Macedonia, to examine how differing degrees of alignment with EU health data and digital health frameworks may shape national approaches. Bulgaria and Romania operate within the directly applicable EU legal framework regarding personal-data protection and digital health development, including the General Data Protection Regulation and the European Health Data Space Regulation, while Serbia and North Macedonia are pursuing different degrees of alignment with the EU but are not subject to these instruments on the same basis.

### 2.2. Data Sources and Materials

Data were collected between January and April 2026 through a structured review of publicly available documentary sources at both national and international levels. The primary data sources included official government and ministry of health websites, national health insurance funds, medicines agencies and other regulatory authorities, legal and policy databases, and scientific publications and reports relevant to medication adherence and digital health implementation. The review was designed as a comparative analysis of heterogeneous documentary evidence rather than as a conventional systematic review of clinical interventions.

For the purposes of source classification, the included materials were divided into three main categories:

(1) Official legal and policy documents, including laws, ordinances, regulations, national strategies, action plans, and governmental policy documents;
(2) Regulatory and institutional sources, including guidance, reports, operational documents, and publicly available information issued by ministries of health, medicines agencies, national health insurance funds, professional organizations, and other competent institutions;
(3) Scientific sources, including peer-reviewed research articles, review articles, and scientific or technical reports identified through database searches and supplementary reference screening, where these sources informed either adherence measurement approaches or the implementation of digital health tools in the four study countries.

Detailed information on the national databases and official institutional sources searched for each country is provided in Supplementary Table S1.

### 2.3. Search Strategy

Searches were conducted independently by the country investigators using country-specific scientific databases, official institutional websites, and legal databases. Searches focused on predefined thematic domains including medication adherence, adherence assessment, pharmaceutical care, electronic prescribing, digital health, reimbursement, and relevant national legislation. The scientific literature was searched in the PubMed, Scopus, and Web of Science databases. The search combined terms related to medication adherence, adherence measurement, digital health, and the names of the four study countries. The following core keywords and their combinations were used: “medication adherence,” “compliance,” “adherence measurement,” “adherence tools,” “pharmacy claims,” “prescription refill,” “electronic monitoring,” “digital adherence technology,” “mobile health,” “mHealth,” “eHealth,” “telemedicine,” “electronic prescription,” “electronic health record,” “therapeutic drug monitoring,” “Bulgaria,” “Romania,” “Serbia,” and “North Macedonia.” Country-specific searches were also conducted using national-language equivalents of the principal search terms where applicable. The literature search covered documents published between January 2010 and April 2026, reflecting the period in which digital adherence tools and related regulatory initiatives became more visible in the study settings. No publication-date restriction was applied to legislation that remained in force during the study period. Because cross-country legal and policy materials vary in availability, terminology, and institutional structure, comparability was treated as analytical rather than formal: the review compared countries only across shared dimensions and did not assume direct equivalence between legal categories or regulatory instruments. Additional evidence from published research on mobile health applications and digital adherence technologies was included to contextualize the regulatory findings and identify how formal frameworks aligned, or failed to align, with reported implementation in practice. To support cross-country comparison, the regulatory review was organized using a common analytical framework covering legal basis, responsible institutions, reimbursement and financing mechanisms, digital infrastructure, and the formal recognition or practical implementation of adherence measurement tools. Each country was reviewed against the same domains to reduce descriptive imbalance and make the basis of comparison explicit.

#### 2.3.1. Inclusion Criteria

The study included documents and data sources that fulfilled the following criteria: (1) addressed national regulatory frameworks, reimbursement arrangements, or implementation of adherence measurement in one of the four countries; (2) referred to digital health systems, telemedicine, or electronic prescribing; (3) were publicly accessible and officially recognized; (4) related specifically to the healthcare systems of North Macedonia, Bulgaria, Serbia, or Romania.

#### 2.3.2. Exclusion Criteria

Documents focused on unrelated clinical outcomes, countries outside the study scope, or non-health-sector uses of digital tools were excluded. Duplicate publications, superseded versions of documents, and sources that did not provide sufficient country-specific information were also excluded. When several versions of the same legal or regulatory document were identified, the most recent applicable or consolidated version was retained.

Initial searches identified 746 potentially relevant sources across the four countries. These comprised 71 official legal and policy documents, 51 regulatory and institutional sources, and 624 scientific publications. All retrieved sources were screened against the predefined eligibility criteria, including relevance to medication-adherence assessment, implementation, regulatory support, reimbursement, and integration into routine healthcare practice.

**Figure 1:**
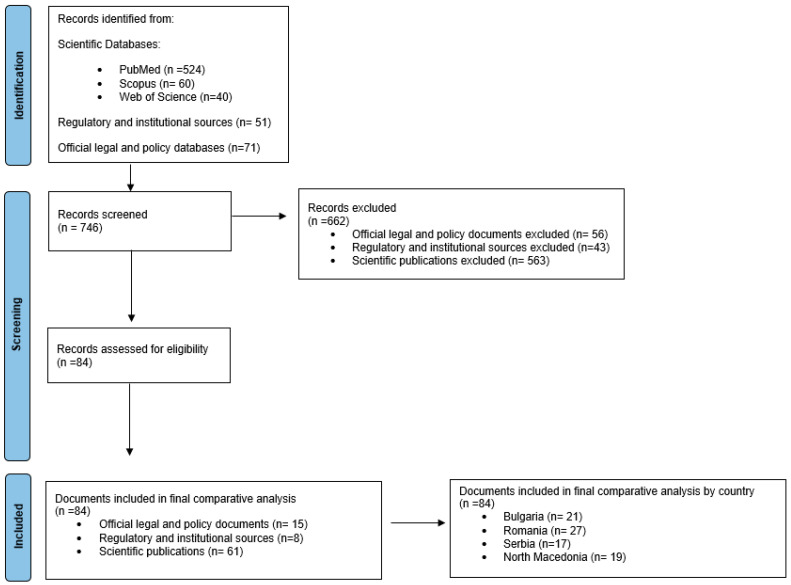
Flow diagram of the identification, screening and inclusion of the documents.

Following screening and eligibility assessment, 662 sources were excluded because they did not address the predefined analytical domains, lacked country-specific information, duplicated information reported elsewhere, or did not meet the source-type and accessibility criteria. A total of 84 documents were included in the final comparative analysis: 15 official legal and policy documents, 8 regulatory and institutional sources, and 61 scientific publications. The included studies comprised 21 sources from Bulgaria, 27 from Romania, 17 from Serbia, and 19 from North Macedonia (Figure 1).

### 2.4. Data Extraction and Classification

The review applied a common extraction grid covering adherence measurement methods, implementation context, and relevant legal and regulatory provisions, so that each country was assessed against the same analytical domains and differences in source availability could be documented rather than treated as equivalent. This approach was used to identify cross-national similarities, differences, and their implications for the use of adherence-related tools across the four healthcare systems.

Data were independently extracted and initially coded by four reviewers, with one reviewer responsible for each country case (Bulgaria, Romania, Serbia and North Macedonia), using a standardized data extraction form developed specifically for this study.

To promote consistency, the form was pilot-tested on a sample of eligible documents and revised before full extraction. Extracted data were subsequently checked for consistency and completeness. Any uncertainties or discrepancies in data extraction or coding were first discussed among all country reviewers to ensure a harmonized interpretation of the predefined extraction criteria across countries. When consensus could not be reached, two senior reviewers independently evaluated the issue and acted as arbiters until a final consensus was achieved.

The extracted information and assigned categories were compared between the reviewers. Disagreements relating to source eligibility, source classification, coding, or interpretation were first discussed between the reviewers involved and, where consensus was not reached, adjudicated through team discussion to arrive at a final coding decision. To make the evidentiary basis of the comparison transparent, all included sources were classified by country and source type. The final evidence base comprised [*N* = 84] unique sources, including [*n* = 21] sources relating to Bulgaria, [*n* = 27] to Romania, [*n* = 17] to Serbia, and [*n* = 19] to North Macedonia (Supplementary Table S2).

### 2.5. Data Analysis

A common search, extraction, classification, and analytical framework was applied to all four country cases to support transparency and comparability. This approach was used to identify cross-national similarities, differences, and their implications for the use of adherence-related tools across the four healthcare systems. The analysis was conducted in two interrelated phases. Phase 1 examined the availability, implementation, and practical use of conventional and digital methods for measuring medication adherence. Phase 2 examined the national legal, regulatory, institutional, reimbursement, and digital health frameworks that enable or constrain the implementation of these methods.

#### 2.5.1. Phase 1: Analysis of Medication Adherence Measurement Methods

In Phase 1, a structured document review was conducted to identify how MA is measured across the four countries. Both conventional and digital measurement approaches were included, such as self-reported questionnaires, pharmacy dispensing and refill data, prescription records, electronic monitoring devices, mobile health applications, and other digital adherence tools. For each country, the analysis focused on the availability of adherence measurement methods and tools, their degree of implementation in routine clinical practice, and their scope of use within the healthcare system. The findings were synthesized and summarized in a comparative matrix presenting adherence measurement methods and tools across the four countries.

#### 2.5.2. Phase 2: Legal and Regulatory Framework Analysis

Phase 2 consisted of a comprehensive review of national legislation, regulations, and official policy documents related to digital health, eHealth and mHealth systems, medication adherence monitoring, and patient data protection. Legal sources included laws on health protection, health insurance, medicinal products and medical devices, electronic health systems, and relevant secondary legislation and national guidelines. The regulatory frameworks of the four countries were systematically compared to assess the extent to which medication adherence measurement tools—particularly digital technologies—are formally recognized, regulated, or supported. Similarities, differences, and regulatory gaps were identified, with particular emphasis on legal provisions that enable or constrain the implementation of digital adherence tools in routine clinical practice. The results were presented in a comparative table that highlighted cross-country similarities and differences, as well as areas lacking regulatory support.

#### 2.5.3. Thematic Content Analysis and Cross-Country Synthesis

All documents were analyzed using thematic content analysis, with coding guided by four predefined domains of medication adherence assessment: (1) electronic databases and pharmacy claims systems; (2) patient self-reported methods; (3) digital technologies and electronic monitoring tools; (4) therapeutic drug monitoring (TDM). For each document, relevant material was extracted and assigned to these domains, then compared across countries using a common analytic framework focused on measurement methods, digital tool implementation, reimbursement arrangements, and regulatory provisions. The reviewers compared coded material across documents within each domain and synthesized themes only after discrepancies in coding or interpretation had been resolved through consensus discussion. This procedure allowed the analysis to move beyond description by applying the same coding structure to each country case and then identifying recurring patterns, divergences, best practices, and unmet regulatory or implementation needs. For each domain, the following aspects were evaluated: level of national implementation; healthcare setting and providers involved; strengths and limitations; regulatory support and reimbursement mechanisms; integration into routine clinical practice. The findings were synthesized descriptively and presented in comparative tables. During the preparation of this manuscript, the authors used ChatGPT 5 and Grammarly for the purposes of translation.

## 3. Results

MA can be assessed using a variety of methods, each characterized by specific advantages, limitations, and areas of application. The selection of an appropriate method depends on the clinical setting, available resources, and the purpose of adherence assessment. Table 1 provides an overview of the most commonly used approaches for measuring medication adherence, together with their strengths, limitations, and typical providers or settings [9,13,14,20,21].

The assessment of medication adherence practices in the four countries revealed considerable differences in the adoption of monitoring methods and the level of regulatory support. The implementation status and regulatory support of adherence measurement methods were categorized using predefined qualitative criteria. “Implemented/Supported” indicates routine availability with evidence of regulatory, policy, reimbursement, or healthcare system support. For patient self-reported methods, “Limited/local use only” indicates non-routine use documented in research studies, pilot projects, or selected healthcare settings; it does not imply validated local-language instruments, nationwide adoption, or formal regulatory support. “Not implemented/Supported” indicates the absence of evidence for routine implementation or formal support. These findings are presented in Table 2.

As shown in Table 2, regulatory support is more developed for electronic prescribing systems and laboratory-based monitoring approaches than for adherence-specific assessment tools and digital adherence technologies. Although all four countries have introduced various digital health reforms, dedicated regulatory frameworks supporting the systematic monitoring of MA remain largely absent. Further country-specific details regarding the implementation and use of individual medication adherence measurement methods are presented in Table 3.

### 3.1. Electronic Database

Electronic prescribing systems and pharmacy claims databases represent one of the primary objective approaches for assessing MA across the analyzed countries. These systems enable the collection of prescribing and dispensing data, creating opportunities for refill-based adherence assessment through indicators such as the Medication Possession Ratio (MPR) and the Proportion of Days Covered (PDC). The Medication Possession Ratio (MPR) represents the proportion of medication supplied over a specified follow-up period. Despite its widespread use, inconsistencies in the calculation of the denominator may introduce variability in adherence estimates and hinder comparisons across studies. The PDC represents the proportion of days within the observation period during which the patient had access to the prescribed medication and is calculated by dividing the number of covered days by the total number of days in the follow-up period. PDC is a standardized metric for assessing medication adherence, with values ranging from 0% to 100%. A threshold of PDC < 80% is widely accepted as an indicator of suboptimal adherence [24,25,26,27].

Among the analyzed countries, Bulgaria, Romania, and Serbia demonstrate the most developed electronic prescribing infrastructures. In Bulgaria, electronic prescribing was introduced as part of the National Health Information System (NHIS) and has been functioning since 2021 [28,29,30,31,32,33,34]. Mandatory electronic prescriptions for reimbursed medicines, antibacterial agents, and selected chronic disease therapies allow real-time tracking of medication dispensing and create a theoretical basis for large-scale adherence monitoring [29]. However, despite the existence of centralized national databases, adherence monitoring is currently limited mainly to administrative and financial control rather than to clinical applications.

Similarly, Romania has implemented several interconnected digital systems, including the Integrated Unique Information System (SIUI), the electronic prescription platform (SIPE), and the electronic health record system (DES) [35,36]. These systems facilitate the recording of prescribed and dispensed medications and provide infrastructure capable of supporting longitudinal adherence analyses [37,38,39,40,41,42]. Nevertheless, limitations related to interoperability, fragmented healthcare databases, and incomplete integration into clinical workflows continue to restrict their effective utilization for routine adherence monitoring [43,44,45].

Serbia has also implemented electronic prescribing and dispensing systems for reimbursed medicines and chronic disease therapies [46,47]. Although these systems theoretically enable adherence evaluation through pharmacy refill indicators, their practical application remains focused predominantly on reimbursement control and regulatory oversight. Limited interoperability between healthcare sectors further constrains integration of adherence-related data into routine clinical decision-making processes.

In contrast, North Macedonia demonstrates a comparatively lower level of implementation regarding electronic adherence monitoring. Although a nationwide e-health infrastructure and electronic prescription systems exist, they are not currently utilized for systematic adherence assessment [48,49,50,51]. Interoperability between healthcare providers and pharmacy systems remains limited, restricting the ability of healthcare professionals to monitor medication-taking behavior comprehensively.

Overall, electronic prescribing and dispensing infrastructure was documented in the mentioned countries, but the reviewed sources provided limited evidence that these databases were routinely used at the national level to calculate standardized adherence indicators or support clinical adherence-monitoring pathways.

### 3.2. Patient Self-Reported Methods

Patient self-reported methods remain one of the most commonly utilized approaches for medication adherence assessment across the analyzed countries. These methods are predominantly implemented through questionnaires, validated adherence scales, and structured patient interviews due to their low cost, ease of administration, and feasibility in both clinical and research settings. Unlike electronically derived measures from prescribing and dispensing systems, self-reported approaches are primarily used for individual-level assessment and are not routinely integrated into national healthcare data systems. Romania demonstrated the highest diversity of questionnaire-based adherence studies, particularly in chronic diseases such as hypertension [52,53,54,55,56], diabetes mellitus [57], hepatitis C [58,59], and respiratory disorders [60,61]. Several studies focused on the translation and validation of adherence tools originally developed in English, whereas fewer investigations aimed to develop population-specific instruments with formally evaluated psychometric properties.

In Bulgaria, self-reported adherence assessment tools are primarily used within academic and research settings [62]. The Morisky Medication Adherence Scale (MMAS) has been translated into Bulgarian and widely applied in observational studies; however, it has not been formally integrated into routine national clinical practice [63,64,65,66,67,68].

Findings from Serbia pointed out that adherence questionnaires are used mainly in research contexts, but also within standardized pharmaceutical services initiated and developed by the Pharmaceutical Chamber of the Republic of Serbia. In addition, the Pharmaceutical Care Network Europe (PCNE) Drug-Related Problems Classification system has been adopted within pharmaceutical care practice [69]. Although not specifically designed for adherence measurement, the system enables pharmacists to identify and document medication-related problems, including non-adherence.

North Macedonia demonstrated the most limited development regarding validated self-reported adherence tools. No psychometrically validated adherence questionnaires in the Macedonian language were identified [70].

Across all countries, self-reported measures remain the dominant methodological approach for adherence evaluation despite their susceptibility to recall bias and overestimation of adherence rates.

### 3.3. Mobile Health (mHealth) Applications

The reviewed sources documented uneven and generally limited formal implementation of digital technologies specifically intended for medication-adherence monitoring across the four countries. Mobile health applications, telemedicine platforms, and remote monitoring systems have emerged primarily within pilot studies, research projects, or broader digital healthcare strategies rather than as standardized clinical tools.

Romania demonstrated the most diverse implementation of digital adherence technologies, including self-developed mobile health applications, remote monitoring systems, and telemedicine-based adherence interventions [71,72]. National digitalization strategies and European Union-supported healthcare modernization initiatives have accelerated the development of digital healthcare infrastructure [36,45]. However, practical implementation remains limited by interoperability issues, insufficient digital literacy, and incomplete integration into routine healthcare workflows [43,44].

In Bulgaria, the National Strategy for e-Health and healthcare digitalization identifies telemedicine and mobile health monitoring as future priorities, particularly for chronic disease management [73]. Despite the existence of the official national e-Health application, no structured adherence monitoring functionalities are currently integrated into the system [74,75].

Serbia has also demonstrated increasing professional awareness regarding digital health technologies. Research among community pharmacists indicates positive attitudes toward mobile applications and digital tools for chronic disease self-management, particularly in diabetes care [76]. Nevertheless, standardized implementation, reimbursement mechanisms, and formal professional training remain limited.

North Macedonia currently demonstrates minimal implementation of digital adherence technologies within routine healthcare practice. No national digital adherence monitoring programs, smart medication devices, or structured mHealth adherence interventions were identified [48].

Electronic monitoring devices, including smart inhalers, electronic pill bottles, compliance cards, and continuous positive airway pressure (CPAP) adherence tracking systems, were identified only sporadically within the analyzed literature. Romania reported limited use of device-based monitoring technologies in respiratory disease management and sleep apnea treatment [77], while Bulgaria, Serbia, and North Macedonia demonstrated minimal evidence of routine implementation or reimbursement of advanced electronic adherence monitoring systems.

### 3.4. Therapeutic Drug Monitoring (TDM)

Therapeutic drug monitoring (TDM) represents an objective biological method for evaluating MA through measurement of drug concentrations or biomarkers in biological samples. Compared with self-reported and pharmacy refill-based methods, TDM provides objective evidence of medication exposure; however, the reviewed evidence documented its availability mainly in selected hospital and laboratory settings and did not identify nationally organized use of TDM specifically for systematic adherence monitoring. In Bulgaria, therapeutic drug monitoring is available primarily within specialized hospital laboratories and institutional healthcare settings [78,79]. Although TDM is used clinically for selected medications requiring narrow therapeutic monitoring, no centralized national adherence monitoring program utilizing TDM has been established.

Similarly, Serbia reported the availability of therapeutic drug monitoring within selected hospitals and specialized laboratories; however, its application for systematic medication adherence assessment remains limited and non-standardized [46,80].

Romanian studies identified several objective adherence assessment approaches involving biological markers and laboratory-based measures; however, these methods were used considerably less frequently compared to questionnaire-based approaches and pharmacy refill analyses [81,82,83]. No evidence of structured national TDM-based adherence monitoring frameworks was identified in North Macedonia.

Table 4 summarizes cross-country patterns in medication adherence policy and implementation across Bulgaria, Romania, Serbia, and North Macedonia. Dedicated national legislation and national adherence strategies were absent in all four countries, and no country reimbursed structured adherence assessment services. Clinical guidelines recommending adherence assessment were identified in Bulgaria and Romania, whereas Serbia and North Macedonia showed only partial guideline support. Validated adherence questionnaires were reported primarily in research settings rather than routine clinical practice across all countries. Electronic prescribing was implemented nationally in all four countries, while pharmacy refill or dispensing data were available in Bulgaria and Romania but limited in Serbia and North Macedonia. Digital health tools and patient portals were more developed in Romania, moderate in Bulgaria, and still developing in Serbia and North Macedonia. Pharmacist involvement was expanding in Bulgaria and Romania but remained limited in Serbia and North Macedonia. Overall, Bulgaria and Romania demonstrated more mature digital infrastructure and greater integration of adherence-related resources into healthcare systems than Serbia and North Macedonia. However, implementation varied across individual adherence assessment methods, as summarized in Table 2.

## 4. Discussion

This comparative analysis examined medication adherence measurement approaches and the regulatory environments shaping adherence-related practices across North Macedonia, Bulgaria, Romania, and Serbia. The findings demonstrate that, despite varying levels of digital health development, medication adherence monitoring remains insufficiently integrated into routine clinical practice across all analyzed countries. This gap implies that current policy priorities are oriented more toward administrative control and reimbursement than toward using adherence data to guide clinical care. As a result, existing digital systems have not yet been translated into standardized, patient-centered adherence support within everyday practice. Similar implementation gaps between digital infrastructure and practical adherence monitoring have been reported in other European healthcare systems [9,16].

Across all four countries, national healthcare policies primarily focus on reimbursement systems, healthcare digitalization, and administrative management rather than on structured adherence assessment and patient-centered adherence interventions. Although the importance of medication adherence for improving therapeutic outcomes, reducing hospitalizations, and lowering healthcare costs is well established internationally [1,15,17], adherence monitoring has not yet become a standardized component of chronic disease management in the region. This finding aligns with the World Health Organization’s recognition of medication adherence as a multidimensional healthcare challenge requiring coordinated policy approaches and multidisciplinary interventions [2,17].

An important contextual factor that should be considered when interpreting these findings is the differing European regulatory environment of the four countries included in this review. Bulgaria and Romania, as European Union Member States, are required to implement common European legislation governing pharmaceutical regulation, data protection, electronic health services, and digital health infrastructure. Although medication adherence assessment is not specifically regulated at the European level, EU policies promoting digital health, patient-centered care, and cross-border interoperability may help explain why Bulgaria and Romania show more developed electronic health infrastructure than Serbia and North Macedonia, potentially creating more favorable conditions for future adherence monitoring. Ongoing initiatives such as the European Health Data Space (EHDS) are expected to further strengthen interoperability and the secondary use of health data, including data relevant to medication adherence monitoring. In contrast, Serbia and North Macedonia, although actively aligning their healthcare legislation with the European acquis as candidate countries, retain greater flexibility in the pace and scope of regulatory implementation. Accordingly, the observed cross-country differences are better understood as arising from the interaction between supranational regulatory alignment and national factors such as healthcare financing, implementation capacity, and professional practice models, rather than from independent national policy choices alone. Nevertheless, EU membership alone does not fully account for the observed variation, as national healthcare priorities, financing mechanisms, implementation capacity, and professional practice models also influence the development of adherence-related services. Serbia and North Macedonia, while progressing in alignment with the EU acquis, still differ from Bulgaria and Romania in the pace of regulatory harmonization, digital health implementation, and integration of pharmaceutical care services; these differences may help explain variation in how adherence measurement is organized and implemented across the four systems.

The findings suggest that medication adherence measurement remains insufficiently integrated into national pharmaceutical policies across the four countries included in this review. Although electronic prescribing systems have become widely available, they are not routinely used to support structured adherence assessment. This pattern is important because it suggests that digitalization alone does not produce clinically meaningful adherence monitoring unless it is accompanied by reimbursement incentives, interoperable data systems, and clearly defined professional responsibilities.

Despite differences in healthcare organization and regulatory development, all four countries demonstrated similar challenges regarding the absence of standardized national approaches to medication adherence assessment. This suggests that improving adherence measurement represents a regional priority rather than an isolated national issue and highlights the need for greater collaboration in developing harmonized regulatory and professional standards.

One of the most important findings of the present analysis is the discrepancy between technological readiness and clinical implementation. Bulgaria, Romania, and Serbia have implemented electronic prescribing systems and pharmacy claims databases, while North Macedonia has partially developed a national e-health infrastructure [28,29,34,35,36,37,38,39,40,41,42,43,44,45,46,47,48,84,85]. These systems theoretically enable objective refill-based adherence assessment using indicators such as Medication Possession Ratio (MPR) and Proportion of Days Covered (PDC), which are internationally recognized approaches for evaluating medication adherence through electronic databases [9,39]. However, in practice, these systems are used predominantly for reimbursement control and administrative purposes rather than for routine adherence monitoring or clinical decision-making. Similar barriers, including limited interoperability, fragmented healthcare systems, and absence of standardized adherence policies, have also been identified in other low- and middle-income healthcare settings [16,56].

Self-reported adherence measures remain the dominant assessment approach across all included countries. Questionnaires and validated adherence scales continue to be widely used because of their low cost, simplicity, and feasibility in both research and clinical settings [9,13]. Romania demonstrated the greatest methodological diversity, including validated questionnaires, prescription record analyses, biological measures, telemedicine interventions, and mobile health applications [52,53,54,55,56,57,59,60,61,71,72,81,82,83]. In contrast, North Macedonia demonstrated the most limited development regarding validated adherence assessment tools and digital adherence monitoring. Nevertheless, despite their widespread use, self-reported methods are associated with important limitations, including recall bias and overestimation of adherence, and none of the analyzed countries has formally integrated standardized adherence questionnaires into routine healthcare practice [15,19].

The study additionally demonstrated limited implementation of digital adherence technologies. Although telemedicine, mobile health applications, and electronic monitoring systems have shown positive effects on adherence and chronic disease outcomes internationally [20,22], their use in the analyzed countries remains fragmented and largely restricted to pilot projects or isolated research initiatives. Romania and Serbia demonstrated comparatively greater institutional readiness and research activity in this area [71,72,76], while Bulgaria and North Macedonia remain at earlier stages of implementation. Importantly, technological availability alone appears insufficient without supportive reimbursement models, interoperability standards, professional training, and integration into routine healthcare workflows.

The COVID-19 pandemic exerted immense pressure on the Romanian healthcare system, and it led to legislative changes which allowed pharmacists to be involved in practices such as COVID testing and vaccination, as well as in digital healthcare practices. Specifically, the Romanian health legislation was amended to include telemedicine, creating a framework that also enabled remote pharmaceutical counseling services, known as telecounseling. These changes highlighted the adaptability of pharmaceutical practice and care in Romania, especially when appropriate regulatory changes and digital infrastructure are present. This further suggests that the structural prerequisites for implementing digital interventions aimed at assessing and improving adherence exist, but specific protocols as well as nationally coordinated efforts in this regard remain insufficient [86].

Therapeutic drug monitoring (TDM) was identified as another underutilized adherence assessment strategy. Although TDM services are available in specialized hospital settings in Bulgaria, Romania, and Serbia [36,46,78], their use remains limited to selected medications and clinical contexts. Similar findings have been reported internationally, where TDM is recognized as an accurate but resource-intensive adherence assessment method with limited applicability for large-scale routine monitoring [9].

An important finding concerns the evolving role of pharmacists in adherence support. International evidence consistently demonstrates that pharmacist-led interventions, including medication counseling, adherence education, medication reviews, and digital support services, can improve adherence and clinical outcomes [15,16]. However, pharmacist-led adherence services remain insufficiently formalized and inconsistently reimbursed across the analyzed countries. Serbia demonstrated comparatively stronger institutional integration through the adoption of the Pharmaceutical Care Network Europe (PCNE) Drug-Related Problems Classification system [87], while studies from North Macedonia and Serbia indicate growing patient expectations and pharmacist readiness regarding expanded pharmaceutical care services [76,88].

The ongoing development of EHDS may provide future opportunities for harmonized adherence monitoring and cross-border digital health integration within the region [89]. However, the observed differences should also be interpreted in relation to the different positions of the four countries within the European regulatory Environment. As European Union Member States, Bulgaria and Romania operate within common EU-level requirements concerning health data governance, interoperability and cross-border digital health services, whereas North Macedonia and Serbia continue to develop their digital health systems and align their national legislation with European Standards through nationally implemented reforms [46,89,90]. This structural distinction may partly explain the comparatively different levels of regulatory maturity and digital health infrastructure identified in the present analysis and suggests that these variations should not be attributed solely to independent national healthcare policies. Nevertheless, EU membership alone does not seem sufficient to ensure the systematic implementation of medication-adherence monitoring. The reviewed evidence indicates that Bulgaria and Romania also lack nationally standardized adherence indicators. Thus, while the EU regulatory environment may provide a framework for digital health development, adherence-specific implementation depends on national legislation, institutional capacity, interoperability and integration into routine clinical practice.

This study has several limitations. The analysis was based primarily on published literature and publicly available policy documents, which may not fully reflect real-world clinical implementation. In addition, heterogeneity between healthcare systems and available evidence limited direct comparability between countries. However, the study provides one of the first comparative regional analyses specifically examining medication adherence monitoring approaches and digital adherence infrastructure across the four healthcare systems examined.

Overall, the findings indicate that the major challenge across the analyzed countries is not the absence of digital infrastructure, but rather the limited translation of existing technological capacity into routine adherence monitoring and patient-centered clinical practice. This observation is consistent with a recent SWOT analysis of artificial intelligence in clinical medicine, which emphasized that technological capability alone is insufficient for successful clinical implementation without coordinated governance, interoperability, workforce readiness, standardized implementation pathways, and supportive regulatory frameworks [91]. Future efforts should focus on improving interoperability, developing standardized adherence indicators, establishing reimbursement models for adherence-support services, and strengthening pharmacist involvement in chronic disease management.

### 4.1. Strengths

One of the main strengths of this research is its regional perspective, which integrates adherence-related practices from several Balkan countries that remain comparatively underrepresented in the international adherence literature. Additionally, the paper includes several dimensions such as clinical, pharmaceutical, and digital health, as well as legislation, rather than focusing exclusively on patient-related factors that influence adherence. The study identifies regulatory, methodological, and infrastructural gaps that should be addressed to support the future development and implementation of standardized medication-adherence assessment and support strategies within existing digital health systems. By including multiple neighboring healthcare systems, common challenges and opportunities regarding adherence assessment and implementation in the mentioned Southeastern European region can emerge.

### 4.2. Limitations

This study has several limitations. First, because the analysis was based on published scientific literature and publicly accessible legal, regulatory, policy, and institutional documents, the findings reflect documented practices and formal system-level support more than actual routine clinical implementation. Informal practices, local institutional procedures, unpublished initiatives, and professional activities not represented in these sources may therefore have been missed.

Second, the available evidence differed across countries. A larger body of indexed research or more accessible institutional documentation may create the impression that adherence-related activity is more developed, when this may instead reflect better documentation rather than stronger implementation. Publication bias and source-availability bias may therefore have influenced the comparison.

Third, digital health technologies and telemedicine systems are rapidly evolving, and the availability or implementation of these tools may change over time.

Fourth, the study included only four countries which limits the generalizability of the findings. The comparative analysis was qualitative and did not include patient-level data or evaluation of clinical outcomes. Finally, differences in national regulations, terminology, and the absence of standardized adherence assessment tools may have affected the comparability of results across countries. An additional limitation concerns the different relationships of the four countries with the European Union. As member states, Romania and Bulgaria are subject to legal and policy requirements concerning areas such as health data protection, digital health interoperability and cross-border electronic health services. Although Serbia and North Macedonia pursue various levels of legislative alignment with European standards, they are not directly bound by these requirements. Thus, some observed differences in regulatory maturity or digital health infrastructure may therefore reflect this structural distinction rather than national policy choices alone.

Despite these limitations, the use of a common comparative framework provides a structured regional overview and identifies areas requiring more detailed investigation.

## 5. Conclusions

From a policy perspective, national health authorities and professional organizations should consider developing standardized frameworks for medication adherence assessment that incorporate validated adherence measurement methods into routine healthcare practice. Greater involvement of pharmacists in adherence monitoring, supported by appropriate professional training, reimbursement mechanisms, and integration within multidisciplinary care models, may strengthen adherence-related services. In addition, digital health systems should evolve beyond administrative functions to enable the systematic use of prescribing, dispensing, and pharmacy claims data for adherence assessment while ensuring interoperability and data protection. Future research should focus on implementation studies evaluating the feasibility, effectiveness, and cost-effectiveness of standardized adherence monitoring strategies, as well as their impact on patient outcomes and healthcare system performance across the four health systems.

The available evidence indicates that subjective self-reported methods remain the predominant approaches described across the four healthcare systems, while the use of electronic databases and real-world data sources appears to be limited, fragmented, and insufficiently integrated into documented routine adherence monitoring.

Although digital infrastructures such as electronic prescribing systems and health information platforms are available in several countries, their application is focused primarily on administrative and reimbursement purposes rather than systematic adherence monitoring.

Regulatory frameworks across the region similarly provide only indirect support for adherence assessment and adherence-promoting services. Overall, the reviewed evidence indicates limited documented and regulatory support for the systematic integration of medication adherence monitoring into routine healthcare practice across the four countries included in this review. Standardized measurement approaches, digital adherence technologies, and pharmacist-led adherence services appear to have been implemented only to a limited extent according to the available literature and policy documents. Across Bulgaria, Romania, Serbia, and North Macedonia, the analysis revealed a shared regional pattern characterized by limited institutionalization of medication adherence measurement and fragmented regulatory support for adherence-related tools.

These findings highlight the need for stronger policy frameworks, harmonized adherence indicators, improved interoperability between healthcare systems, and greater integration of digital health technologies to support systematic medication adherence monitoring in the four countries examined, while providing insights that may be relevant for other countries with similar healthcare and regulatory contexts.

## Figures and Tables

**Table 1:** Methods for measuring medication adherence.

Method	Strengths	Limitations	Provider/Setting
Electronic database (pharmacy claims database)	Easy to use, inexpensive, non-invasive; supports retrospective adherence assessment [9,13]	Suggests medication supply without assurance of administration; may overestimate adherence [9,13]	Pharmacy systems, health insurance funds
Patient self-reported methods (questionnaires, visual analog scales)	Easy applicability in clinical practice; cost-effective and efficient; evaluates adherence and persistence [19]	Subjective; influenced by recall and reporting bias; overestimation of adherence [19]	Healthcare professionals (physicians, pharmacists); clinical setting
Digital adherence technologies (mHealth applications, electronic monitoring devices)	Objective data (sensor-based devices only); real-time monitoring and reminders; provides additional information on adherence patterns; enables remote monitoring [9,21]	Many mHealth applications rely on patient self-report and are therefore susceptible to reporting bias; No direct evidence of actual ingestion; high costs; limited routine use in clinical practice [9,21]	Patients at home; healthcare providers via mHealth apps and telemedicine platforms
Therapeutic Drug Monitoring (TDM)	Accurate and objective; provides direct evidence of drug concentration; can detect over- or underdozing [9,19]	Invasive and costly; measures adherence at a single time point; not suitable for all medications; inter-individual variability [9,19]	Clinical laboratories; hospital setting

**Table 2:** Comparative overview of medication adherence measurement methods, implementation status, and regulatory support.

Method	North Macedonia	Bulgaria	Serbia	Romania	Regulatory Support
Electronic databases/pharmacy claims systems	◐	✓	✓	✓	✓ Indirect support through e-prescribing, reimbursement and health information system legislation
Patient self-reported methods	◐	◐	◐	◐	✗ No specific national regulations or standardized adherence assessment requirements were identified in North Macedonia, Bulgaria and Romania; ◐ in Serbia, adherence assessment is standardized nationally for specific conditions within standardized pharmaceutical services
Digital technologies (mHealth applications, electronic monitoring devices)	✗	◐	◐	◐	◐ Digital health and telemedicine strategies exist, but adherence-specific regulation is limited
Therapeutic Drug Monitoring (TDM)	◐	◐	◐	◐	✓ Regulated through laboratory and clinical practice legislation, but not specifically for adherence monitoring

Abbreviations: ✓ Implemented/Supported; ◐ Limited/local use only; no national policy or standardized requirement; ✗ Not implemented/Supported.

**Table 3:** Country-specific data sources and methods used for medication adherence assessment.

Method	North Macedonia	Bulgaria	Serbia	Romania
Electronic databases/pharmacy claims systems	E-prescribing available; limited use for adherence monitoring.	NHIS collects pharmacy claims data; mainly administrative and reimbursement use.	Mandatory e-prescribing and dispensing for reimbursed medicines.	SIUI and SIPE systems enable collection of prescribing and dispensing data.
Patient self-reported methods	Questionnaires used mainly in research; no nationally validated routine tool.	MMAS translated and used in research settings; not integrated into routine practice.	PCNE classification adopted; adherence assessment standardized nationally for specific conditions within standardized pharmaceutical services	Multiple validated questionnaires used in research; no routine national implementation.
Digital technologies (mHealth applications, electronic monitoring devices)	No national adherence monitoring programs identified.	National eHealth application available; no structured adherence monitoring functionality.	Telemedicine and digital health strategy exists; no adherence monitoring devices implemented or reimbursed.	Mobile applications and telemedicine interventions reported; no national standardization.
Therapeutic Drug Monitoring (TDM)	Limited availability in hospital settings.	Available in specialized hospital laboratories.	Available in selected hospitals and laboratories.	Available in hospitals; not routinely used for adherence monitoring.

Abbreviations: MMAS, Morisky Medication Adherence Scale; NHIS, National Health Information System; PCNE, Pharmaceutical Care Network Europe; SIUI, Integrated Unique Information System; SIPE, Electronic prescription platform; TDM, Therapeutic Drug Monitoring.

**Table 4:** Comparative overview of medication adherence measurement methods and regulatory frameworks across the four countries.

Domain	Bulgaria	Romania	Serbia	North Macedonia	Comparative Summary
National legislation addressing medication adherence	Partial	Partial	Limited	Limited	No country has dedicated legislation specifically regulating medication adherence assessment.
National adherence strategy	No	No	No	No	Common regional gap.
Clinical guidelines recommending adherence assessment	Yes	Yes	Partial	Partial	Guidelines exist, but recommendations vary in scope and implementation.
Validated adherence questionnaires (MMAS, MARS, BMQ, etc.)	Research use	Research use	Research use	Research use	Mainly applied in research rather than routine clinical practice.
Pharmacy refill/dispensing data	Available	Available	Limited	Limited	Availability depends on national digital infrastructure.
Electronic prescribing	National	National	National	National	Implemented in all countries, but not routinely used for adherence monitoring.
Digital health tools/patient portals	Moderate	Advanced	Developing	Developing	Different levels of digital maturity.
Pharmacist involvement	Expanding	Expanding	Limited	Limited	Clinical pharmacy services remain heterogeneous.
Reimbursement of adherence services	No	No	No	No	None of the countries reimburses structured adherence assessment.
National implementation level	Partial	Partial	Limited	Limited	Similar implementation challenges across the region.
Main regulatory gap	Lack of standardized framework	Lack of standardized framework	Limited regulatory support	Limited regulatory support	Regional need for harmonized regulatory guidance.

## Data Availability

No new data were created or analyzed in this study. Data sharing is not applicable to this article.

## Supplementary Materials

The following supporting information can be downloaded at https://www.mdpi.com/article/10.3390/healthcare14152419/s1: Supplementary Table S1. National databases and official information sources consulted during the search process; Supplementary Table S2. The composition of the final evidence base.

## Abbreviations

The following abbreviations are used in this manuscript:

CPAP	Continuous Positive Airway Pressure
EHDS	European Health Data Space
HCPs	Healthcare Professionals
MA	Medication Adherence
MMAS	Morisky Medication Adherence Scale
MPR	Medication Possession Ratio
NHIS	National Health Information System
PCNE	Pharmaceutical Care Network Europe
PDC	Proportion of Days Covered
SIUI	Integrated Unique Information System
SIPE	Electronic Prescription Platform
TDM	Therapeutic Drug Monitoring
