# Supplementary material for: Medication Adherence Measurement in Southeast Europe: A Comparative Analysis of Assessment Methods, Digital Health Implementation, and Regulatory Frameworks
Source: Healthcare (Basel). 2026 Aug 6;14(15):2419. doi: 10.3390/healthcare14152419 (PMC13464670; doi:10.3390/healthcare14152419)
Supplement: Supplementary file 1 [file healthcare-14-02419-s001.zip › healthcare-4431316-supplementary.pdf]

Supplementary Table S1: National databases and official information sources consulted during the search process

Country	National databases / official sources searched
**Bulgaria**	Ministry of Health; Bulgarian Drug Agency (BDA); National Health Insurance Fund (NHIF); Bulgarian Pharmaceutical Union (BPhU); National Centre of Public Health and Analyses; National Health Information System (NHIS)
**Romania**	Ministry of Health; National Agency for Medicines and Medical Devices (ANMDMR); National Health Insurance House (CNAS); Romanian College of Pharmacists
**Serbia**	Ministry of Health; Medicines and Medical Devices Agency of Serbia (ALIMS); Republic Health Insurance Fund (RFZO); Pharmaceutical Chamber of Serbia
**North Macedonia**	Ministry of Health; Agency for Medicines and Medical Devices (MALMED); Health Insurance Fund; Pharmaceutical Chamber of North Macedonia

Supplementary Table S2: The composition of the final evidence base

**Country**	**Official legal and policy documents (laws, ordinances, regulations, governmental policy documents)**		**Regulatory and institutional sources (guidance, reports, operational documents)**		**Scientific publications (research articles, review articles)**		**Total included:**
	Screened	*Included*	*Screened*	*Included*	*Screened*	*Included*	
**Bulgaria**	N=20	N=7	N=10	N=3	N=97	N=11	N=21
**Romania**	N=4	N=1	N=9	N=3	N=404	N=23	N=27
**Serbia**	N=27	N=2	N=14	N=1	N=49	N=14	N=17
**North Macedonia**	N= 20	N=5	N=18	N=1	N=74	N=13	N=19
**Total**							**N=84**
